# Ultrasound monitoring of skeletal muscle wasting and relation to nutritional intervention in critically ill patients: MUScleNut study

**DOI:** 10.1186/s40635-025-00823-y

**Published:** 2025-12-01

**Authors:** Catarina Rosa Domingues, Simão C Rodeia, Ana Rita Francisco, Laura Santos, Carolina Cerca, Madalena Costa, Vera Pinto, Philip Fortuna, Ana Brito-Costa, Luís Bento

**Affiliations:** 1 Intensive Care Medicine Department, Hospital de São José, Unidade Local de Saúde São José, Lisboa, Portugal; 2 iNOVA4Health, NOVA Medical School, Faculdade de Ciências Médicas, NMS, FCM, Universidade NOVA de Lisboa, 1169-056 Lisboa, Portugal; 3 Centro de Estatística E Aplicações (CEAUL), Faculdade de Ciências, Universidade de Lisboa, Lisboa, Portugal; 4 Departamento de Estatística E Investigação Operacional (DEIO), Faculdade de Ciências, Universidade de Lisboa, Lisboa, Portugal; 5 Nutrition Unit, Hospital de São José, Unidade Local de Saúde São José, Lisboa, Portugal; 6 Laboratório de Nutrição, Faculdade de Medicina, Universidade de Lisboa, Lisboa, Portugal; 7 Gabinete de Análise Epidemiológica E Estatística Do Centro de Investigação, Unidade Local de Saúde São José, Lisboa, Portugal; 8 CHRC, NOVA Medical School, Faculdade de Ciências Médicas, NMS, FCM, Universidade NOVA de Lisboa, Lisbon, Portugal

**Keywords:** Ultrasound, Muscle wasting, Nutrition, Intensive care, Intensive care unit acquired weakness

## Abstract

**Background:**

Critically ill patients frequently experience profound skeletal muscle (SM) wasting, to which early detection and effective clinical management remain significant challenges. Ultrasonography (US) provides early objective information about SM compared with usual functional tests. The characteristics of the optimal nutritional support are controversial. This observational study aimed to characterize the SM changes through US in the first week after Intensive Care Unit (ICU) admission and to evaluate the potential interference factors with a focus on nutritional support.

**Results:**

A total of 95 patients (age 55.7 ± 16.01 years, 70.5% male) were included. All the ultrasound SM measures tendentially reduced after admission: quadriceps muscle layer thickness (QMLT) 10.03% (0.38 ± 0.73 cm), rectus femoris cross-sectional area (RF-CSA) 10.48% (0.50 ± 1.38 cm^2^), RF pennation angle (RF-PA) 0.94 ± 4.14 º, RF echogenicity (RF-EG) 1.05 ± 22.33 in echo-intensity gray scale and RF shear wave elastography (RF-SWE) 0.13 ± 1.25 m/s and 3.96 ± 28.10 kPa. A significant association between nutritional risk at baseline and SM changes (QMLT 0.194, *p* = 0.079 and RF-CSA 0.25, *p* = 0.027) was observed and confirmed in a linear regression model (1.257 and *p* = 0.011). No significant associations were found between SM changes and nutritional support.

**Conclusion:**

Present findings demonstrate a marked reduction in the SM ultrasound measures evaluated in the first week after ICU admission, mainly in patients at nutritional risk. More evidence on optimal nutritional strategies to attenuate SM wasting is warranted.

**Supplementary Information:**

The online version contains supplementary material available at 10.1186/s40635-025-00823-y.

## Introduction

Skeletal muscle (SM) wasting occurs early and acutely after the Intensive Care Unit (ICU) admission, mainly in the first week [1] contributing to the development of ICU-acquired weakness (ICU-AW) [2], which is characterized by an extensive loss of muscle mass, function and quality, most pronounced among patients with multiorgan dysfunction [3, 4]. It is estimated that half of critically ill patients develop ICU-AW [5, 6], leading to worse clinical outcomes, specifically difficult mechanical ventilation weaning [7–9] and impaired rehabilitation [10, 11], both associated with a prolonged ICU and hospital length of stay (ICU-LOS) and higher mortality [12].

SM wasting is a complex and multifactorial condition associated with anabolic resistance and impaired utilization of dietary substrates for muscle synthesis, parallel to increased protein catabolism. Reduced peripheral blood flow induced by vasoactive medications and prolonged immobilization experienced by critically ill patients is also an important contributor [13].

Measuring and monitoring SM in the ICU is still challenging. There has been a growing interest in ultrasonography (US) for monitoring SM changes, as it is a non-invasive, free of radiation tool readily available in the ICU that could be performed at the patient’s bedside [14]. It is valid and reliable [15], comparable to expensive gold standard imaging methods [16–18], and has been shown to be a promising alternative in providing early objective information about SM changes compared with performance-based and strength-based tests, namely medical research council score (MRC), traditionally used for ICUAW diagnosis [19, 20] when the patient is already awake and able to collaborate in the test [21]. Thus, different US measures have been proposed, namely quantitative measures such as quadriceps muscle layer thickness [22, 23] and cross-sectional area [12, 24], but also innovative measures suggested to be representative of muscle quality such as pennation angle [25], echogenicity [26] and shear wave elastography [27].

The evidence about the association between nutrient delivery and muscle wasting is controversial, with a lack of physiological studies and robust clinical trials with adequate randomization between groups [28, 29]. Optimal nutritional support during the ICU length and after, combined with early physical rehabilitation has been suggested to have a synergic effect in attenuating SM wasting [30, 31]. However, the definition of “optimal nutritional support” needs to be elucidated concretely, as well as the energy and protein targets and the timing to reach them.

The present study aims to characterize the SM changes in the first week after ICU admission and evaluate the potential interference factors, focusing on nutritional support.

## Materials and methods

### Ethics statement

The Research ethics committee at Hospital de São José, Unidade Local de Saúde São José has approved the present study.

### Study design and patients

This observational prospective study aimed: 1) to characterize the SM changes in the first week after ICU admission and 2) to evaluate the potential interference factors, with a focus on nutritional support, non-nutritional caloric infusions, cumulative fluid overload, ICU-AW diagnosis and demographic, clinical and nutritional characteristics.

Between April 2023 and January 2024, all the adult patients (age ≥ 18 years) admitted in the Medical Emergency Unit (UUM) and Extracorporeal Membrane Oxygenation Unit (ECMO-ICU) with expected ICU-LOS of ≥ 7 days were screened for eligibility. Patients were excluded if: (i) hospitalization length > 72 h prior to ICU admission; (ii) pre-existing neuromuscular pathology, musculoskeletal lesions of the lower limbs or amputation; (iii) any previous condition that could compromise the patient’s mobility; (iv) imminent death or potential organ donor admission; (v) impossibility to perform US (e.g., impossibility of positioning, access to the measurement sites or problems in the acquisition and analysis of images), according to the protocol proposed in the present study; (vi) patients eating by mouth; (vii) inability to obtain informed consent from the patient or carer.

All the patients were followed throughout their ICU-LOS, with the data collection focusing on two study time-points, in the first 72 h (skeletal muscle assessment 1, SMA 1) and 1 week (SMA 2) after ICU admission. All the patients were screened for the start of medical nutrition therapy (MNT) in the first 24 h of admission, as well as for the start of physiotherapy in the first 48 h of ICU admission, according to the nutrition and physiotherapy protocols in practice in the UUM and ECMO-ICU.

### Data collection

Data were recorded from an approved electronic medical record system (EMR) (B-Simple ® PatientCare ®). and consisted of demographic, clinical, nutritional data and ultrasound images: (i) demographic and clinical data were collected throughout the ICU-LOS and included patient age, gender, reason for ICU admission (medical or surgical), comorbidities, severity of illness through sequential organ failure assessment (SOFA) and acute physiology and chronic health evaluation II (APACHE II) scores, type and duration of invasive mechanical ventilation (IMV), type and duration of ECMO support, dose and duration of renal replacement technique (RRT), dose and days of propofol, glucose, citrate, lactate, ICU-LOS, in-hospital LOS, readmission rate, ICU and hospital mortality and 90-day mortality; (ii) nutritional data were collected in the first week after ICU admission, at two study time-points (SMA 1 and 2) and included data from nutritional assessment, namely actual body weight (BW), height and body mass index (BMI). Dried BW was preferred, particularly in patients in post-resuscitation and patients with renal failure. Adjusted body weight was used for obese patients (≥ 30 kg/m^2^) according to ESPEN guidelines [32]. These anthropometric parameters were preferentially measured, instead of reported or estimated. Additionally, nutritional screening tools were performed, such as Nutritional Risk Screening 2002 (NRS 2002) [33], comprising information about BW loss, low BMI, reduced food intake, severity of illness, and NUTRIC score [34], completing the information from NRS 2002 with information about severity of illness scores (SOFA and APACHE II), number of comorbidities, hospital LOS and inflammation. Nutritional data also included energy expenditure (EE) estimation, using weight-based equations proposed by the European Society of Clinical Nutrition and Metabolism (ESPEN) [32] considering the energy targets of 70% of 20 to 25 kcal/kg BW/day in the acute phase and 25 to 30 kcal/kg BW/day in the late phase with a non-protein-energy caloric dose of 60% of the total energy requirements as carbohydrates and 40% as fat, according to ESPEN, due to the absence of indirect calorimetry, recommended as the gold standard method for EE assessment and protein requirements estimation considering a target of 1.3 g/kg BW/day for general critically ill patients, with particularities for obese, trauma and dialyzed patients. Nutritional intervention followed the nutritional protocol in practice in UUM and ECMO-ICU, which are based on ESPEN guidelines, defining the start of MNT in the first 24 h after admission after hemodynamic, respiratory and electrolyte balance stabilization and progressive achievement of energy and protein targets (in 3 days), and includes clear instructions regarding the MNT route, type and formulation, volume and rate, micronutrient supplementation, fluid intake and how to act in cases of gastrointestinal intolerance. To standardize the data collection, it was defined to acquire data from EMR up to 23h59 of the day before any study time-point, respectively, SMA1 corresponds to nutritional data of the first 72 h and SMA2 to the first week after ICU admission. Energy and protein balances were calculated based on the differences between the prescribed and administered energy and protein amounts. The non-nutritional caloric infusions (propofol, glucose, citrate, lactate) were included; (iii) ultrasound images were obtained at two study time-points (SMA 1 and SMA 2). Quantitative and qualitative measures of the rectus femoris muscle (RF) and vastus intermedius muscle (VI) were obtained, respectively, RFVI thickness or quadriceps muscle layer thickness (QMLT) and RF cross-sectional area (RF-CSA) and RF pennation angle (RF-PA), RF echogenicity (RF-EG) and RF shear wave elastography (RF-SWE). All the SM ultrasound measures with the exception of RF-SWE were done with the Philips Lumify ® portable system using a two-dimensional (4–12 MHz), 5.6 cm linear transducer, or Venue 2.5 GE ® system using a two-dimensional (6–13 MHz), curvilinear transducer, choosing the one that allowed the best measurement of the desired measure and visibility (depth and laterality) depending on the patient's anthropometric characteristics, given that the variability of the measurements using two types of transducers has been previously assessed [35]. RF-SWE was done with the ACUSON Sequoia Ultrasound System, Siemens Healthineers ® using a high-frequency (15 MHz) linear transducer. All the ultrasound images were collected by two trained investigators (CRD and ABC), with the inter-observer variability evaluated as very good in a pilot cohort of 58 patients (63 pairs of measures; ICC = 0.965 (0.943 – 0.979) *p* < 0.01). Images were captured directly on the US system and subsequently exported without any adjustments to a computer for further analysis. Image analysis was done using the image analysis software ImageJ (National Institutes of Health, Bethesda, Maryland; https://imagej.nih.gov/ij/). All measures were performed in the quadriceps, choosing the available leg considering the cannulas placement in case of ECMO support. Three measurements were performed, and the arithmetical average was used in the final analysis. The ultrasound image acquisition followed a protocol created for the present study (Supplementary material), which standardized the patient position, anatomic landmarks, transducer placement, as well as image analysis.

### Statistical methodology

#### Variable classification

The following variables were considered continuous: age, SOFA, APACHE II, BW, BMI, duration of IMV, ECMO support and RRT, dose and days of propofol, glucose, citrate and lactate, ICU-LOS and in-hospital LOS, readmission rate, NRS 2002 and NUTRIC scores, energy and protein requirements, prescription, intake and cumulative fluid balance and all the SM ultrasound measures (QMLT, RF-CSA, RF-PA, RF-EG, RF-SWE).

The following were treated as categorical or binary: gender (male/female), admission category (medical/surgical), ICU-AW diagnosis (yes/no), IMV, RRT and ECMO support (yes/no) and mortality outcomes (ICU and hospital mortality and 90 days mortality). For exploratory or subgroup analyses, continuous variables were categorized when appropriate (e.g., NRS ≥ 5 vs < 5) to improve clinical interpretability or manage small sample sizes.

#### Statistical analysis

All statistical analyses were conducted using IBM SPSS Statistics (version 28.0, IBM Corp., Armonk, NY, USA). Descriptive statistics were used to summarize the study population. Continuous variables are presented as mean ± standard deviation (SD), and categorical variables as absolute numbers and percentages. The distribution of continuous variables was assessed using the Shapiro–Wilk test, complemented by visual inspection of Q–Q plots and boxplots. Where assumptions of normality were not met, nonparametric methods were used.

For within-subject comparisons of SM ultrasound measures between baseline (SMA1) and follow-up (SMA2), paired t-tests were applied to normally distributed differences (e.g., QMLT, RF-PA), while Wilcoxon signed-rank tests were used for non-normally distributed variables (e.g., RF-CSA, RF-EG, RF-SWE). Differences were calculated as SMA2—SMA1. *p*-values below 0.05 were considered statistically significant and annotated in Figure 2. Between-group comparisons of continuous variables (e.g., SM change by sex or ICU-AW diagnosis) were analyzed using independent samples t-tests, with Levene’s test used to assess equality of variances. Non-parametric tests were considered when parametric assumptions were violated.

Correlation analyses were performed to assess associations between continuous variables, using Pearson correlation coefficients for normally distributed data and Spearman’s rank correlation otherwise. Statistically significant correlations (*p* < 0.05) were marked with asterisks in the correlation matrix (Fig. 3). Pairwise deletion was used to handle missing data.

Linear regression models were developed to identify predictors of SM change, using QMLT_dif and RF CSA_dif (difference between SMA2 and SMA1) as dependent variables. Univariable models were used to evaluate associations with NRS 2002, ICU-AW diagnosis, cumulative fluid balance until SMA2, and ECMO support (Tables 5 and 6). Results are reported as regression estimates (β), standard errors, *t*-values, 95% confidence intervals (CI), and *p*-values. Model fit was assessed using F-statistics, R^2^, and adjusted *R*^*2*^, and assumptions were verified via residual plots.

Associations between categorical variables were evaluated using Chi-square tests; Fisher’s exact test was applied when expected cell counts were below five. Results are presented with observed and expected counts, proportions, and *p*-values.

## Results

**Fig. 1:**
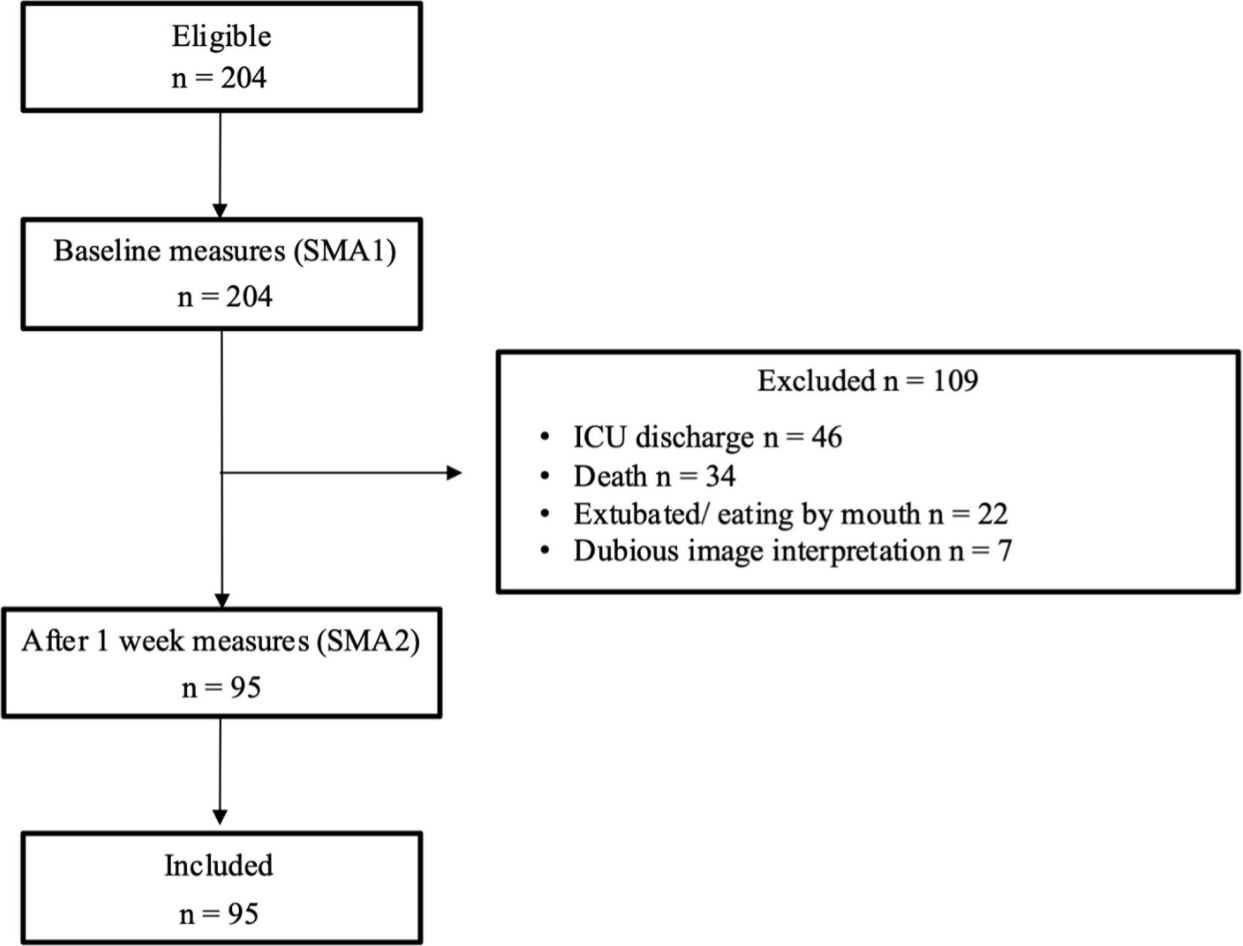
Study flow diagram

Over a 9-month period, 201 patients were admitted on UUM and ECMO-ICU and considered eligible for the study, of which 95 were included (Fig. 1). Table 1 describes the patients’ demographic and clinical characteristics. Overall, the mean age was 55.70 ± 16.01 years with 70.5% (*n* = 67) male. The patients were admitted to the ICU for surgical or medical causes, 33.7% (*n* = 32) or 63.2% (*n* = 60), respectively. Among patients admitted for medical reasons, the most common causes were respiratory failure, sepsis, shock, acute kidney injury, and other related conditions, accounting for a total of 49.5% (*n* = 47). Additional causes included cardiorespiratory arrest 4.2%, trauma 25.3%, and neurocritical disease 9.5%. Among patients admitted for surgical causes, 7.4% were urgent and 1.1% elective, with 3.2% (*n* = 3) of patients having a non-defined admission cause. Severity of illness scores were applied with mean SOFA (8.65 ± 0.44) and APACHE II (20.11 ± 0.83) scores at baseline. Over the ICU LOS, most patients (*n* = 88) required invasive mechanical ventilation (IMV) for an average of 11.92 ± 8.86 days. Renal replacement therapy (RRT) was administered to 33 patients for 12.96 ± 13.14 days, and 17 patients underwent ECMO (14 ECMO-VV and 3 ECMO-VA). ICU-acquired weakness (ICU-AW) was diagnosed in 11 patients (12.5%), based on the Medical Research Council (MRC) sum score, using the standard diagnostic cut-off of < 48 out of 60, assessed at the first moment the patients were able to cooperate with the MRC test.

**Table 1:** Patients’ demographic and clinical characteristics

		All patients (*n* = 95)
Age (years)	Mean ± SD	55.70 ± 16.01
Gender (% male)	% (n)	75 (67)
Admission category		
Medical	% (n)	63.2 (60)
Surgical	% (n)	33.7 (32)
SOFA	Mean ± SD	8.65 ± 0.44
APACHE II	Mean ± SD	20.11 ± 0.83
Duration of organ support (days)		
IMV	Mean ± SD	14.07 ± 13.08
RRT		11.92 ± 8.86
ECMO		12.96 ± 13.14
ICU AW (% yes)	% (n)	12.5 (11)
ICU LOS (days)	Mean ± SD	17.2 + 10.91
ICU mortality rate	% (n)	23.2 (22)
90-day mortality	% (n)	29.5 (28)

*SOFA* Sequential Organ Failure Assessment, *APACHE II* Acute Physiology and Chronic Health Evaluation II, *IMV* invasive mechanical ventilation, *RRT* renal replacement therapy, *ECMO* extracorporeal mechanical ventilation, *ICU-AW* ICU-acquired weakness, *ICU LOS* ICU length of stay

Table 2 summarizes the patients’ nutritional status at admission. Most patients were classified as being at nutritional risk based on the NRS 2002, with 61.1% (*n* = 58) scoring 3 and 25.3% (*n* = 24) scoring 4. A small proportion, 4.2% (*n* = 4), were not assessed. A notable subgroup was identified as having higher nutritional risk, defined by NRS 2002 scores ≥ 5 in 9.5% (*n* = 9) and NUTRIC scores ≥ 5 in 45.3% (*n* = 43).

**Table 2:** Patients’ nutritional status characteristics

		All patients (*n* = 95)
Anthropometric characteristics		
Weight (kg)		78.08 ± 15.52
Height (cm)	Mean ± SD	1.69 ± 0.10
BMI (kg/m2)		27.04 ± 4.93
Nutritional risk screening		
NRS 2002		
Score 3–5	% (n)	86.4 (82)
Score ≥ 5		9.5 (9)
NUTRIC		
Score < 5	% (n)	54.7 (52)
Score ≥ 5		45.3 (43)

*BMI* body mass index, *NRS 2002* Nutritional Risk Screening 2002, NUTRIC score

MNT was started at 23.1 ± 25.8 h. Table 3 describes the nutritional support in SMA 1 and SMA2. The mean energy and protein intake until baseline SMA (SMA 1) was 313.11 ± 474.68 kcal/day, including 147.18 ± 312.10 kcal/day from MNT, 165.92 ± 328.92 kcal/day from non-nutritional caloric infusions, and 5.96 ± 13.36 g/day of protein. After one week (at SMA 2), the mean energy and protein intake were 1215.69 ± 430.91 kcal/day, with 960.33 ± 455.59 kcal/day derived from MNT and 255.35 ± 299.98 kcal/day derived from non-nutritional caloric infusions and 55.06 ± 75.14 g/day, respectively, revealing an increase of MNT support from the acute to late phase of critical illness. Comparing with the energy and protein estimated requirements at SMA 2, 1113.39 ± 286.33 kcal/day and 94.86 ± 17.99 g/day, respectively, the MNT support was 15% above and 51% below these targets.

**Table 3:** Nutritional support in SMA1 and SMA2

		SMA 1 (baseline)	SMA2 (after 1 week)
Nutritional requirements Energy (kcal/day) Protein (g/day)	Mean ± SD	1055.03 ± 263.47 94.86 ± 17.99	1113.39 ± 286.33 94.86 ± 17.99
Energy intake (kcal/day) MNT Non-nutritional caloric infusions	Mean ± SD	313.11 ± 474.68 147.18 ± 312.10 165.92 ± 328.92	1215.69 ± 430.91 960.33 ± 455.59 255.35 ± 299.98
Protein intake (g/day)	Mean ± SD	5.96 ± 13.36	55.06 ± 75.14
Δ energy (%)^1^ Δ protein (%)^2^	Mean ± SD	− 31.91 ± 47.11 − 91.47 ± 20.16	+ 15.83 ± 51.45 − 51.92 ± 24.50

^1^Δ energy and ^2^Δ protein, respectively, represent the variation between nutritional intake and requirements during the specific interval of SMA1 (from ICU admission to 72 h) or SMA2 (from SMA1 to 1 week after ICU admission)

*MNT* medical nutrition therapy

The mean ICU-LOS was 17.2 ± 10.91 days with an ICU-mortality rate of 23.2% (*n* = 22) and a 90-day mortality rate of 29.5% (*n* = 28) (Table 4).

**Table 4:** SM changes in SMA1 and SMA2

		SMA 1 (baseline)	SMA2 (after 1 week)
QMLT (cm)	Mean ± SD	3.10 ± 1.08	2.73 ± 0.99
RF-CSA (cm)	Mean ± SD	2.82 ± 1.46	2.31 ± 1.33
RF-PA (º)	Mean ± SD	9.40 ± 3.90	8.27 ± 2.95
RF-EG (gray scale)	Mean ± SD	78.65 ± 35.04	77.1 ± 33.65
RF-SWE (m/s)	Mean ± SD	3.05 ± 1.36	2.88 ± 1.04
RF-SWE (Kpa)	Mean ± SD	33.79 ± 31.38	28.62 ± 21.16

Abbreviations: *QMLT* quadriceps muscle layer thickness, *RF-CSA* rectus femoris cross sectional area; *RF-PA* rectus femoris pennation angle, *RF-EG* rectus femoris echogenicity, *RF-SWE* rectus femoris shear wave elastography

Baseline skeletal muscle (SM) measurements were obtained at day 2.12 ± 0.90 after ICU admission (SMA1) and repeated after one week, at day 7.59 ± 0.91 (SMA2). The mean baseline (SMA1) skeletal muscle (SM) characteristics were as follows: QMLT 3.10 ± 1.08 cm, RF-CSA 2.82 ± 1.46 cm^2^, RF-PA 9.40 ± 3.90º, RF-EG 78.65 ± 35.04 (gray scale), RF-SWE 3.05 ± 1.36 m/s and 33.7 ± 31.38 kPa. By SMA2, all SM parameters showed a numerical decrease. There was a general tendency toward reduction in all US measures between SMA1 and SMA2. Specifically, QMLT and RF-CSA showed mean reductions of 10.03% (0.38 ± 0.73 cm) and 10.48% (0.50 ± 1.38 cm^2^), respectively. For the other SM variables, the average changes were as follows: RF-PA –0.94 ± 4.14°, RF-EG –1.05 ± 22.33 in echo-intensity gray scale, and RF-SWE –0.13 ± 1.25 m/s and –3.96 ± 28.10 kPa. Paired comparisons confirmed statistically significant reductions in RF-CSA (*p* = 0.027, Wilcoxon signed-rank test) and RF-PA (*p* = 0.046, paired *t*-test). The reduction in QMLT showed a trend toward significance (*p* = 0.068, paired t-test), while changes in RF-EG (*p* = 0.508), RF-SWE in m/s (*p* = 0.605), and RF-SWE in kPa (*p* = 0.242) were not statistically significant. These findings support a consistent pattern of muscle loss in the early ICU stay, particularly in thickness and cross-sectional area measures, with less marked or variable change in muscle echogenicity and stiffness.

**Fig. 2:**
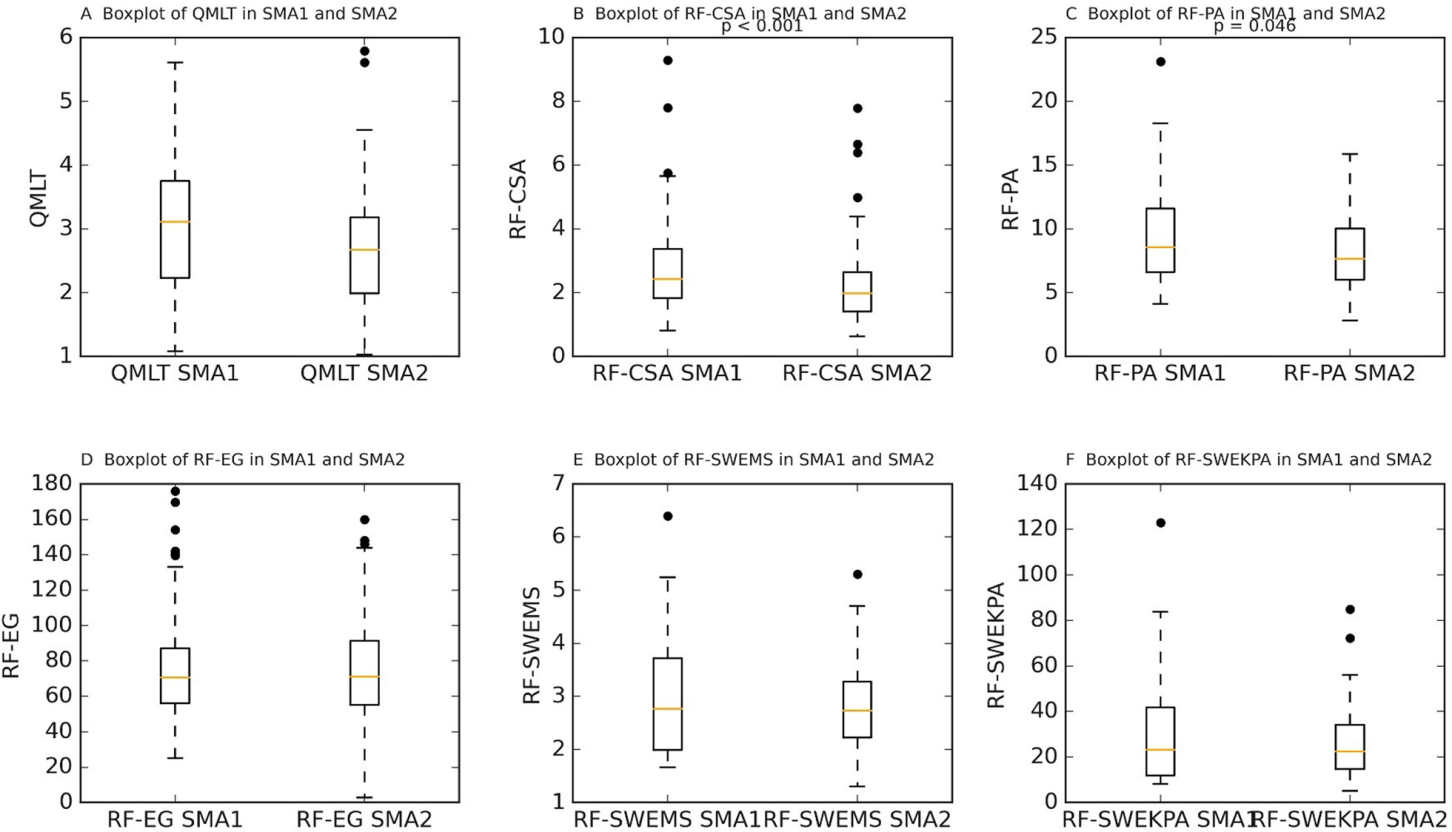
SM changes in SMA1 and SMA2. The figure presents changes in SM US measures: QMLT (cm), RF-CSA (cm^2^), RF-PA (degrees), RF-EG (grayscale units), RF-SWE (Kpa and m/s). Each boxplot shows the median (orange line), interquartile range (IQR), whiskers extending to 1.5 × IQR, and outliers (circles). Statistical comparisons between SMA1 and SMA2 were performed using a paired t-test for QMLT, RF-PA, and RF-SWEMS, and the Wilcoxon signed-rank test for RF-CSA and RF-EG; p-values are indicated above each boxplot to highlight only the significant changes

These results are graphically depicted in Fig. 2, which presents boxplot comparisons of all US measures between SMA1 and SMA2.

Quantitative skeletal muscle changes (QMLT, RF-CSA) between SMA1 and SMA2 were assessed in relation to nutritional risk using the NRS 2002 at baseline. Patients classified at high nutritional risk (NRS ≥ 5) showed greater muscle loss, with a Spearman correlation coefficient of 0.194 (*p* = 0.079) for QMLT and 0.25 (*p* = 0.027) for RF-CSA. While the correlation with QMLT was not statistically significant, the association with RF-CSA was confirmed in a linear regression model, with a regression coefficient of 1.257 (*p* = 0.011; Table 5). This indicates that each point increase in NRS 2002 was associated with a 1.257 cm^2^ greater loss in RF-CSA. The differences were not significant for RF-PA and RF-EG. All the nutritional support and non-nutritional infusions were not associated with SM changes in either categorical or continuous measures, even when they were corrected for weight.

**Table 5:** Linear regression analysis of the association between NRS and changes in RF-CSA

RF-CSA_dif	Estimate	St error	*t*	*P* >|*t*|	CI	
NRS 2002	1.257	0.427	2.942	0.011	0.334	2.180
R-squared	0.353					
Adjusted R-squared	0.400					

F-statistic 8.652; p-value (F-test) 0.011

The dependent variable is RF-CSA_dif, representing the change in rectus femoris cross-sectional area between SMA2 and SMA1. The independent variable is the NRS 2002 score at baseline. The regression coefficient (“Estimate”) represents the expected change in RF-CSA_dif for each one-point increase in NRS 2002. The 95% confidence interval (CI) and corresponding p-value are also reported. A statistically significant positive association was found between NRS 2002 and RF-CSA_dif (*p* = 0.011), indicating greater muscle loss in patients with higher nutritional risk

*RF-CSA* rectus femoris cross sectional area, *RF-CSA_dif* difference between SMA 2 and SMA 1, *NRS 2002* Nutritional Risk Screening 2002

**Fig. 3:**
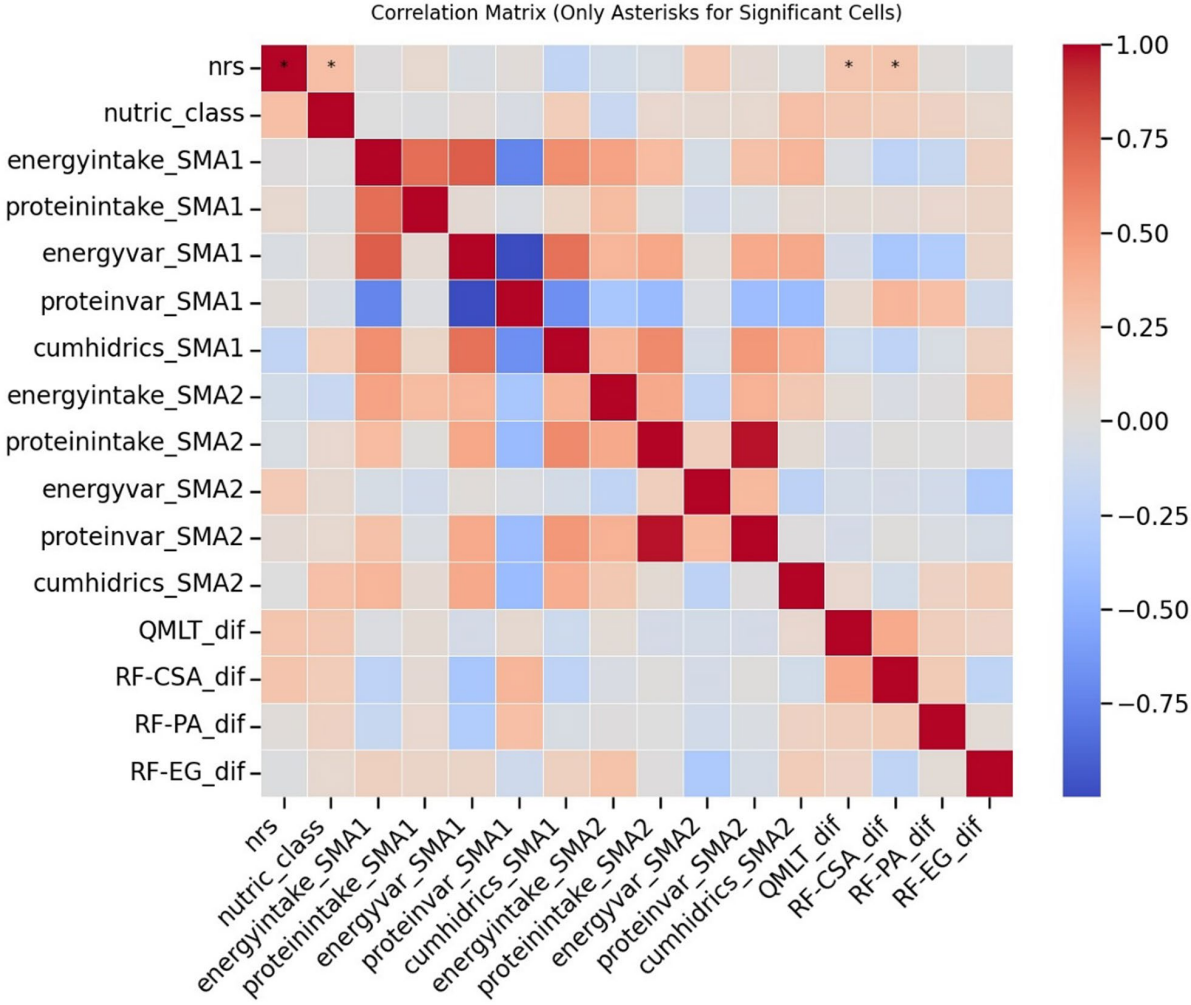
Correlation matrix. Correlation matrix (Pearson’s R coefficients) between skeletal muscle (SM) changes and nutritional support variables. Differences in SM ultrasound measures (e.g., QMLT_dif, RF-CSA_dif) reflect the change from SMA1 to SMA2. Asterisks (*) indicate statistically significant correlations at a threshold of *p* < 0.05. The matrix highlights a significant negative association between NRS 2002 and changes in QMLT and RF-CSA, while no associations were found between nutritional support and SM changes. *QMLT_dif* muscle layer thickness difference between SMA 2 and SMA 1, *RF-CSA_dif* rectus femoris cross-sectional area difference between SMA 2 and SMA 1;

ICU-AW diagnosis was negatively associated with SM changes from SMA1 to SMA2. Patients diagnosed with ICU-AW experienced greater quadriceps muscle layer thickness (QMLT) loss (mean difference: -0.188 cm, *p* = 0.094). Although this difference was not statistically significant, it was supported by a mean group difference in QMLT loss of –0.468 cm (*p* = 0.068; 95% CI: –0.121 to 0.868) between patients with and without ICU-AW. This suggests a potential discrepancy between clinical diagnosis and ultrasound evaluation, as several patients without ICU-AW showed notable QMLT decline (Fig. 3).

As shown in Table 6, ICU-AW was the strongest predictor of QMLT change in univariable linear regression (estimate = –0.756, *p* = 0.003). Two additional predictors were also identified: cumulative fluid balance until SMA2, which averaged 3.61 ± 4.39 L (*β* = 2.654E–5, *p* = 0.007), and ECMO support (estimate = –0.510, *p* = 0.018).

**Table 6:** Linear regression models predicting change in QMLT

Model	Variable tested	Estimate	St error	t	*P* >|*t*|	CI	
1	ICU-AW	− 0.756	0.198	− 3.817	0.003	− 1.192	− 0.320
2	Cumhydrics_sma2	2.654E−5	0.000	3.337	0.007	0.000	0.000
3	ECMO	− 0.510	0.183	− 2.790	0.018	− 0.913	− 0.108

Model 1 F-statistic 12.528; *p*-value (F-test) 0.003

Model 2 F-statistic 5.532; *p*-value (F-test) 0.007

Model 3 F-statistic 7.785; *p*-value (F-test) 0.018

Each model includes QMLT_dif as the dependent variable, representing the difference in quadriceps muscle layer thickness between SMA1 and SMA2. Independent variables are: Model 1: ICU-AW (Intensive Care Unit Acquired Weakness; categorical: yes/no); Model 2: Cumhydrics_sma2 (cumulative fluid balance until SMA2; continuous); Model 3: ECMO support (Extracorporeal Membrane Oxygenation; categorical: yes/no). The regression coefficient (“Estimate”) reflects the expected change in QMLT_dif for a one-unit increase in the independent variable. In Models 1 and 3, this corresponds to the presence of ICU-AW or ECMO, respectively

Model 1 (ICU-AW): Patients with ICU-AW had, on average, a 0.756 cm greater reduction in QMLT compared to those without ICU-AW (*p* = 0.003, 95% CI: − 1.192 to − 0.320)

Model 2 (Cumulative Fluid Balance): For each unit increase in fluid balance (in liters), there was an estimated increase of 2.654E-5 cm in QMLT, through the small magnitude suggests minimal clinical impact. This association was statistically significant (*p* = 0.007)

Model 3 (ECMO): Patients supported with ECMO had a 0.510 cm greater reduction in QMLT compared to those without ECMO (*p* = 0.018, 95% CI: − 0.913 to − 0.108)

*QMLT* quadriceps muscle layer thickness, *QMLT_dif* difference between SMA1 and SMA2, *Cumhydrics_sma2* cumulative fluid until SMA2, ECMO, extracorporeal membrane oxygenation

## Discussion

Early screening of SM after ICU admission may help elucidate further the mechanisms associated with SM loss and other metabolic and nutritional disturbances and, thus, contribute to the optimization of clinical strategies.

The main findings of our study are: (1) a reduction in all the SM ultrasound measures, QMLT, RF-CSA, RF-PA RF-EG and RF-SWE in the first week after ICU admission. (2) Nutritional risk assessment at baseline may be relevant in identifying patients more predisposed to lose SM throughout the first week after ICU admission when evaluated through US. (3) SMA through US may provide a more rigorous evaluation of SM changes that are not evident at the MRC test performed in ICU-AW diagnosis. (4) No significant associations were found between SM changes and nutritional support in the first week after ICU admission.

The findings on SM changes between SMA1 and SMA 2 were in accordance with evidence from previous studies in critically ill patients, which described a 20% reduction in QMLT (4) and a 12.5–16.8% reduction in RF-CSA at day 7 after ICU admission [1, 4], that continued to decrease to day 10. Hayes K et al. found an increased reduction (19.2%) of RF-CSA at day 10 after ICU admission in critically ill patients with ECMO support [36], that continued to decrease to day 20 (30.5%), and a significant decrease from baseline to day 20 in all the other SM ultrasound measures evaluated, QMLT, RF-EG and RF-PA, which was in accordance with our findings for QMLT that reduced in mean percentage 24.11 ± 7.18 compared with 10.95 ± 12.10 in patients without ECMO support, suggesting that these patients may have a worse SM wasting compared with the general critically ill patient population. RF-CSA results were inconsistent (22.74 ± 56.35 and 25.56 ± 26.94 mean percentages, respectively, for patients with and without ECMO support), which could be due to the increased fluid retention in these patients. Sabatino A et al. evaluated the SM changes in critically ill patients with acute renal injury, of which 70% required RRT, observing a 15% reduction in RF and VI thickness each [37], which concurred with our findings (QMLT, and RF-CSA reduced in mean percentage 14.30 ± 13.79 and 29.40 ± 26.87, respectively, compared with 14.7 ± 15.87 and 26.24 ± 31.63 for patients without RRT support), suggesting that the former may have a worse SM wasting.

The observed reductions in SM ultrasound measures between SMA1 and SMA2 must be interpreted with consideration of potential confounding factors, particularly fluid balance and edema. Prior research has shown that muscle thickness may initially increase due to fluid resuscitation, with thickness values still elevated upon ICU discharge compared to preadmission levels. In our study, US were initiated on day 2 of ICU stay (SMA1), potentially capturing the resolution of fluid overload alongside muscle loss. This is especially relevant for subgroups such as ECMO and RRT patients, who are prone to significant fluid shifts during early critical illness. Thus, while the mean percentage reductions in QMLT and RF-CSA (24.11% and 22.74%, respectively, in ECMO patients) are consistent with prior studies, we acknowledge that these may reflect not only muscle catabolism but also the resolution of edema. Edema likely contributes to variability and limits precision in quantifying true SM wasting during early ICU stay.

The relationship between SM wasting and worse clinical outcomes is well established. Lee ZY et al. concluded that for every 1% loss in QMLT there is an increase of 5% in 60-day mortality over the first week of critical illness [12]. No association with mortality could be drawn from our findings, which we think could be due to the high heterogeneity of critical illness conditions.

RF-PA has been described as a SM ultrasound measure potentially representative of SM strength [38], such that a greater RF-PA allows more parallel sarcomere fibers to be packed in a given CSA, necessary to produce strength. Although the reduction in this SM ultrasound measure was not statistically significant (*p* = 0.068), a decreasing trend was observed, which may be suggestive of SM atrophy and consequently associated with worse clinical outcomes, which concurred with findings by Lakenman PLM et al. [39]. Echogenicity has been reported to increase with prolonged ICU LOS [26], as a consequence of histological alterations, such as myofiber necrosis and fascial inflammation, creating a hyperechoic appearance (white) [3, 40]. However, our findings were not consistent, demonstrating a non-significant slight decrease in the mean RF-EG difference between SMA 1 and SMA 2 (78.65 ± 35.04 and 77.1 ± 33.65). The inconsistency in EG results was shared in previous studies [4]. Shear wave elastography (SWE) is a novel SM ultrasound measure that has been suggested to provide a quantitative measurement of SM shiftiness, enabling an early objective evaluation of muscle quality, and has demonstrated a higher reliability with echogenicity and computed tomography [27]. The only previous study focused on the longitudinal assessment of SM SWE in critically ill patients [41], evaluated the diaphragm muscle and observed a decrease in diaphragmatic SWE in the first week after ICU admission (Day 0 and Day 3, 16.4 ± 4.3 kPa and 14.1 ± 3.1 kPa, respectively), suggesting that the decreased stiffness is consistent with the development of muscle injury and weakness, which concurred with our findings in RF muscle (SMA 1 and SMA 2, 33.79 ± 31.38 and 28.62 ± 21.16, respectively).

Several authors have investigated the association between US and other functional and strength-based tests that support ICU-AW diagnosis. Weiqing Zhang et al. assessed the diagnostic accuracy of SM changes evaluated through US and MRC test, revealing a good performance between the two approaches, with the US method being performed at early stages of critically illness [19]. Although the aim of our study was not to compare the US with ICU-AW diagnose method, our findings suggest that some patients who were not diagnosed with ICU-AW in their ICU-LOS had a profound SM loss in the first week after ICU admission, evident by QMLT reduction (mean difference: − 0.188 cm, *p* = 0.094), supported by a mean group difference in QMLT loss of –0.468 cm (*p* = 0.068; 95% CI: –0.121 to 0.868) between patients with and without ICU-AW, revealing that US may be essential in early identifying myopathic patients and planning interventions. Also, a relationship between SM ultrasound measures and nutritional risk screening has been made. Mukhopadhyay A et al. found that patients with high modified NUTRIC score (> 5), considered at high nutritional risk, at baseline lost significantly more SM compared to the low (≤ 5) group (the adjusted ratio for the high versus low group of the geometric mean was 0.58 (95% CI 0.46–0.75) and 0.61 (95% CI 0.49–0.77) for the right and left RF muscles, respectively) [42], which concurred with our findings (QMLT and RF-CSA mean differences between SMA 1 and SMA 2 were significantly greater in the group of patients at higher nutritional risk, 0.194, *p* = 0.079 and 0.25, *p* = 0.027, respectively).

International critical care nutrition guidelines [34, 43] recommend a wide range of energy (20–30 kcal/BW/day, following a “permissive underfeeding” rationale of providing 70% of energy requirements in the acute phase of critical illness) and protein (1.2–2 g/BW/day, with higher protein targets for some patient groups, namely obese, trauma, dialysate patients). Recent systematic reviews and meta-analyses (SRMA) and clinical trials [44–46] have investigated the pertinence of higher protein interventions on improving functional outcomes, with no evident effect on clinical and functional outcomes and finding deleterious effects in some patient groups. In the present study we assessed the potential interference of MNT in SM changes, based on the ESPEN guideline, with both continuous and categorical variables, finding no significant results. Also, the potential later effects of MNT in SM (non-evident in the first week of ICU LOS) are still unknown. It has been demonstrated that amino acid conversion is improved with time, allowing an increase in whole body protein production only after the early period of the acute phase, even increasing further in the post-acute phase [47, 48].

Our study has several strengths: (1) we only included patients with < 72h in-hospital LOS, enabling us to observe the trend of muscle loss over the ICU LOS starting from the usual muscle status of the patient; (2) the diverse potential interference factors investigated and the focus on nutritional intake. To the best of our knowledge this is the first study with the aim to evaluate the association between the longitudinal US assessment of SM and nutritional intake in critically ill patients.

Additionally, although no associations between SM loss and mortality could be drawn from our sample, this may be partially attributed to selection bias and the heterogeneous nature of critical illness. Echogenicity, which may reflect tissue composition and edema, did not show consistent trends in our cohort, possibly due to these fluid-related effects. Regarding functional outcomes, ICU-AW diagnosis was based on MRC testing, but only a subset of patients (*n* = 11; 12.5%) could be evaluated due to the need for adequate cognitive status, which inherently excluded deeply sedated or non-cooperative patients. These methodological considerations, especially the influence of edema and limitations in physical assessment, should be accounted for in future longitudinal studies to improve the accuracy of SM monitoring and its implications for outcomes.

Some limitations should be acknowledged: (1) the relatively small sample size may limit statistical power, precision, and external validity; (2) the results may not be generalizable to other muscle groups, as only the quadriceps was assessed; (3) we did not perform strength or physical function-based assessments to further explore their relationship with skeletal muscle (SM) changes; (4) nutritional requirements were estimated using predictive equations rather than direct methods (e.g., indirect calorimetry or urinary nitrogen losses), which may not reflect actual individual needs and could have influenced the interpretation of SM changes; (5) outcome data, including mortality, were not digitally recorded for the full cohort of 204 screened patients, as the exclusion process occurred before the research database was created. As such, we were unable to analyze mortality rates in the excluded population or fully assess the selection bias introduced by early discharge, death, or extubation prior to the second US assessment.

## Conclusion

Present findings about ultrasound monitoring of SM wasting over the first week after ICU admission demonstrate a marked reduction in all the quantitative and qualitative measures evaluated, highlighting the relevance of this method in early identifying SM changes.

Further studies investigating the interference of the nutritional intake over the ICU LOS and SM wasting trend and testing different nutritional strategies to attenuate SM wasting are warranted.

## Supplementary Information

Additional file 1.

## Data Availability

The datasets used and analyzed during the study are available from the corresponding author on reasonable request.

## Abbreviations

SM: Skeletal muscle
US: Ultrasonography
ICU: Intensive Care Unit
QMLT: Quadriceps muscle layer thickness
RF: Rectus femoris
VI: Vastus internus
RF-CSA: Rectus femoris cross-sectional area
RF-PA: Rectus femoris pennation angle
RF-EG: Rectus femoris echogenicity
RF-SWE: Rectus femoris shear wave elastography
ICU-AW: Intensive care unit acquired weakness
ICU-LOS: Intensive care unit length of stay
MRC: Medical research council
SMA: Skeletal muscle assessment
MNT: Medical nutrition therapy
EMR: Electronic medical record
SOFA: Sequential Organ Failure Assessment
APACHE II: Acute Physiology and Chronic Health Evaluation II
IMV: Invasive mechanical ventilation
RRT: Renal replacement therapy
BW: Body weight
BMI: Body mass Index
NRS 2002: Nutrition risk screening 2002
ESPEN: European Society of Clinical Nutrition and Metabolism
