# Supplementary material for: Ultrasound monitoring of skeletal muscle wasting and relation to nutritional intervention in critically ill patients: MUScleNut study
Source: Intensive Care Med Exp. 2025 Dec 1;13:122. doi: 10.1186/s40635-025-00823-y (PMC12669447; doi:10.1186/s40635-025-00823-y)
Supplement: Supplementary file 1 — Additional file 1. [file 40635_2025_823_MOESM1_ESM.docx]

**Supplementary material**

The images acquisition for SMA over the ICU-LOS followed the next protocol, developed for the present study:

1. Patients positioning and ultrasonography measurement method:
   1. The patient was positioned supine with a pillow under the head and legs and knees extended and in neutral rotation.
   2. The transducer was placed directly on the skin, perpendicular to the skin surface and using water-soluble transducer gel. The images were triply obtained by performing minimum pressure (considering only the pressure inherent to the probe weight on the skin surface).
   3. Measurements were performed only on one leg, chosen according to the ease of performing the measurement (e.g. leg opposite to the ECMO cannulation and free of medical devices). For patients with ECMO cannulation in both legs the venous cannulation leg was chosen for patients on ECMO-VA and the leg with better access was chosen for patients on ECMO-VV.
   4. Measuring points were defined by palpation of the anatomical regions and measured with a tape measure. Measuring points were marked on the skin surface using a dermatographic pen respectively (from proximal to distal):
      1. The ½ point of the distance between upper midpoint of the anterior face of the iliac spine and midpoint of the upper border of the patella.
         - 1. From this point, in the transverse plane, was possible to carry out the measurement of the maximum thickness of QML (Figure I, Sup Material), corresponding to RF and VI thicknesses, considering the image where it is possible to visualize the greatest distance between the cortical zone of the femur and the upper aponeurosis of the RF. Also, this point was used to determine RF echogenicity (RF-EG) and measure RF shear wave elastography (RF-SWE), as it provides a large enough image of RF to accommodate the region of interest for both these analyses.
           2. From this point, in the longitudinal plane, was possible to measure the RF pennation angle (RF-PA) (Figure II, Sup Material), which is the angle of insertion of the muscle fibers of the RF into the inferior aponeurosis (could be identified as the separation zone between the RF and the VI).
         1. The ⅔ point of the distance between the upper midpoint of the anterior face of the iliac spine and the midpoint of the distance between the two femoral epicondyles (medial and lateral).
            1. From this point, in the transverse plane, was possible to carry out the measurement of the RF cross-sectional area (RF-CSA) (Figure III, Sup Material), as it is the only point where it is possible to obtain complete visibility of the circumference limits of the RF. In some cases, particularly in the case of obese patients, was impossible to have the complete visibility of the circumference limits of the RF using linear transducers, so only the images were obtained using a curvilinear transducer.
   5. QMLT was calculated in centimeters in the middle of the ultrasound image at a 90-degree angle.
   6. RF-CSA and RF-PA were measured in centimeters squared and degrees, and were calculated by manually tracing the inner echogenic line of the RF fascia and trancing the insertion of the muscle fibers of the FR into the inferior aponeurosis, respectively, using the image analysis software ImageJ (National Institutes of Health, Bethesda, Maryland; <https://imagej.nih.gov/ij/>).
   7. RF-EG was determined using the quantitative grayscale analysis of ImageJ. One square region of interest (ROI) was determined by placing the largest possible square within the anatomic boundaries of RF. Mean and SD echogenicity of this ROI were expressed (values between 0, black, and 255, white).
   8. RF-SWE was determined directly in the ACUSON Sequoia Ultrasound System, Siemens Healthineers ®, where a SWE quality panel (left) and color map SWE image (right) were simultaneously displayed on USG monitor (Figure IV, Sup Material). Two circular regions of interests (ROIs) were determined by placing the largest possible circles within the anatomic boundaries of RF. Then the mean of ROIs in each of three SWE image were considered to represent the stiffness (kPa) and shear wave velocity (m/s) of evaluated muscle.
   9. The accuracy of the technique and interpretation, as well as the inter-observer variability was evaluated in a pilot cohort previously to the present study.

**
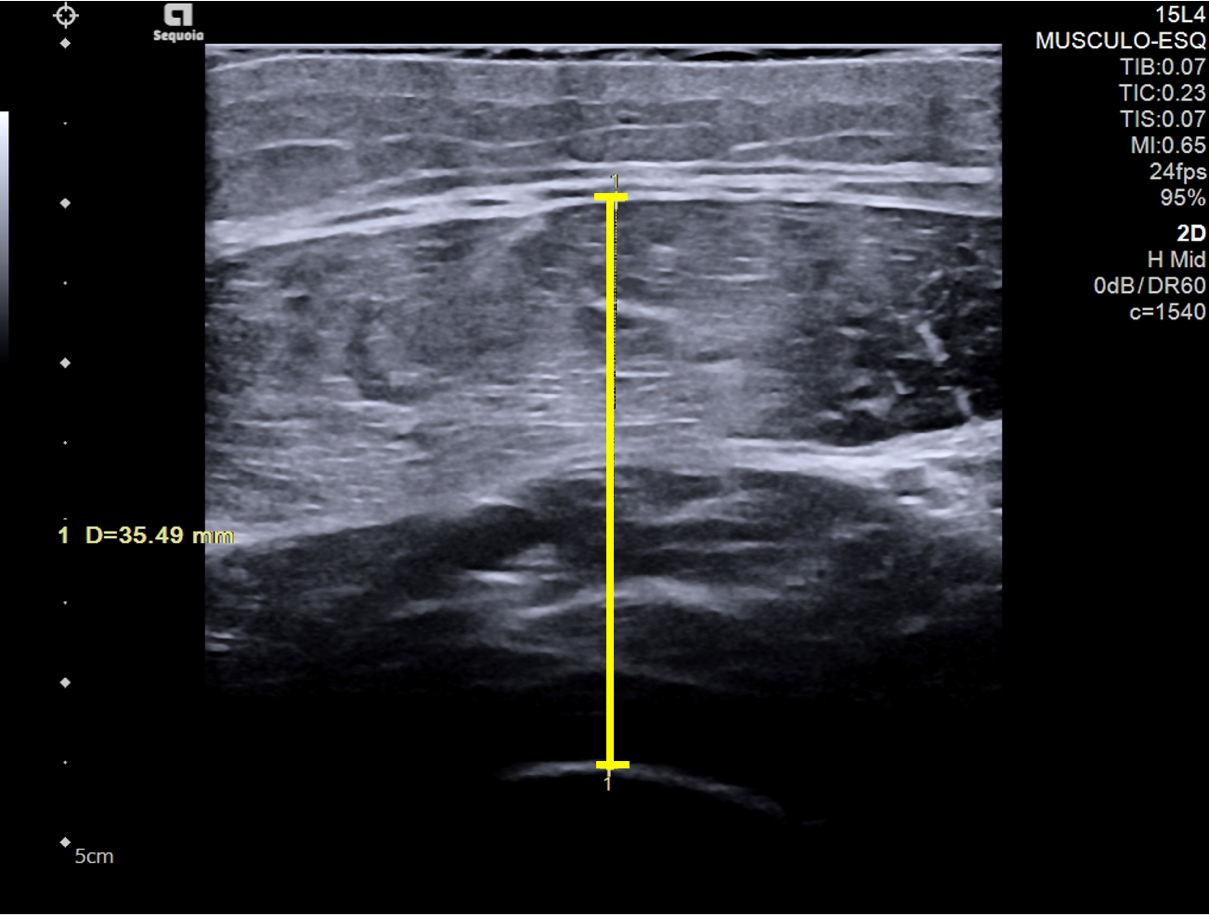
Figure I, Sup Material. Ultrasound image of the QMLT.**

The figure represents the measurement of maximum thickness of QML (RF + VI) in millimeters (then calculated in centimeters), in the middle of the ultrasound image at a 90-degree angle. For purpose of the present Supplementary material and to allow the best quality of the images, the SM ultrasound measurements were all done with ACUSON Sequoia Ultrasound System, Siemens Healthineers ® using a high frequency (15 MHz) linear transducer.

**
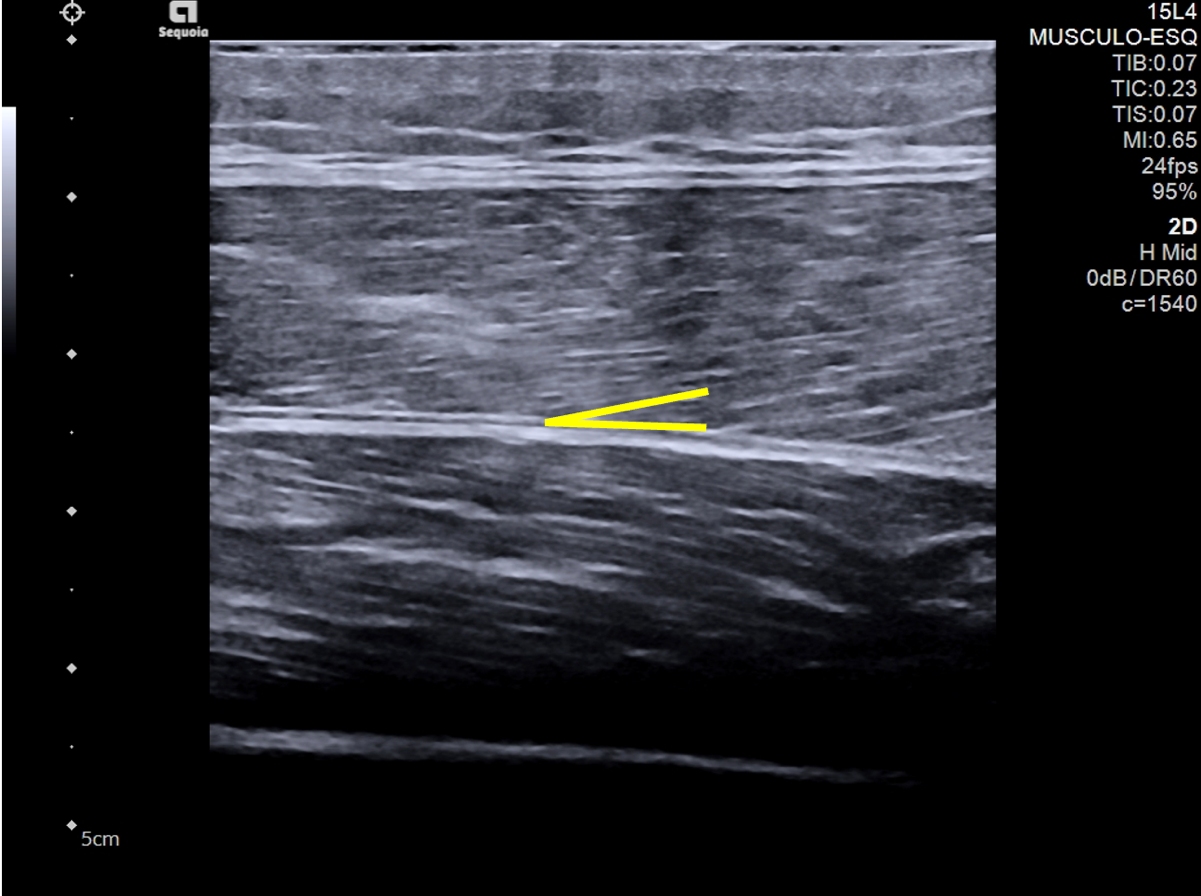
Figure II, Sup Material. Ultrasound image of the RF-PA**.

The figure represents the manually tracing of RF-PA, the angle of insertion of the muscle fibers of the RF into the inferior aponeurosis (identified as the separation zone between the RF and the VI). The measurement of RF-PA was done using the image analysis software ImageJ.


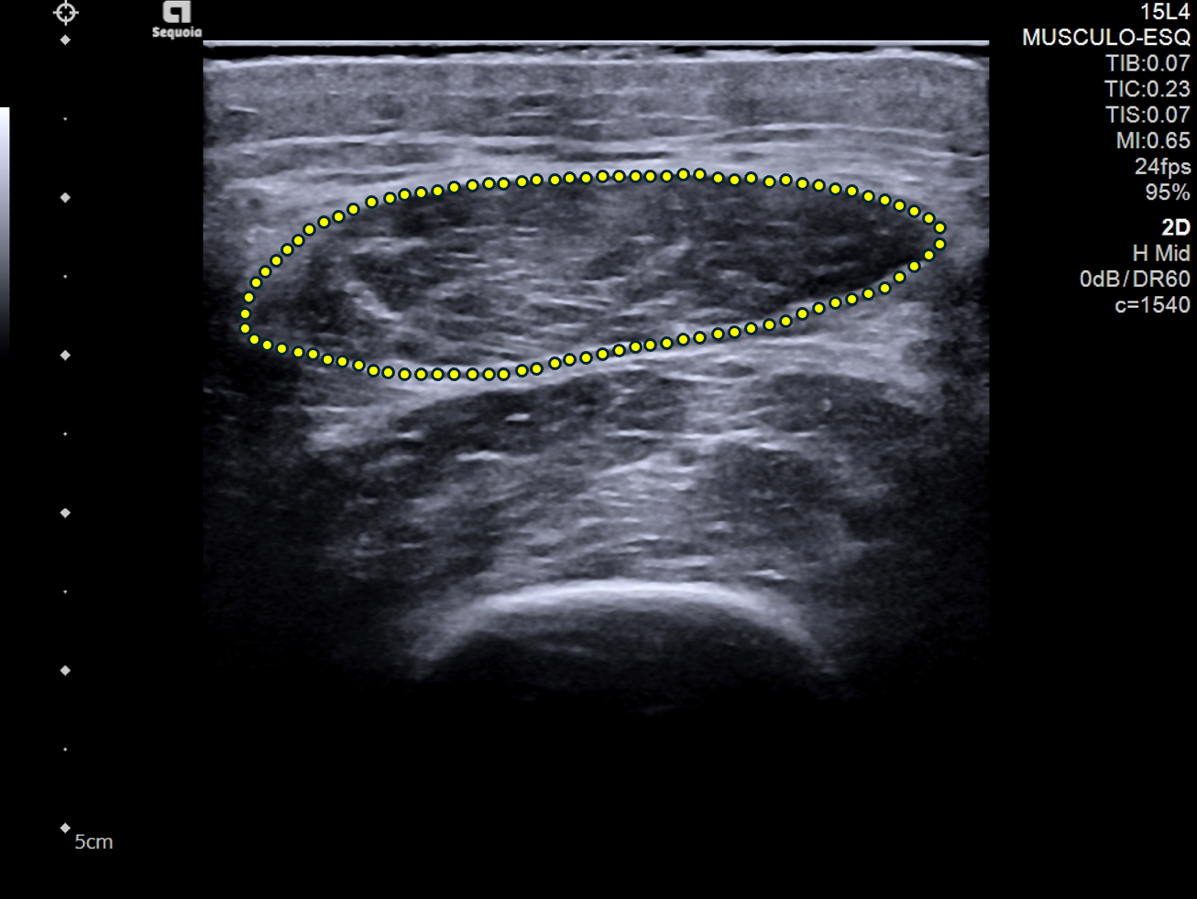
**Figure III, Sup Material. Ultrasound image of the RF-CSA**.

The figure represents the manually tracing of RF-CSA in the middle of the ultrasound image. The measurement of RF-CSA was done using the image analysis software ImageJ.


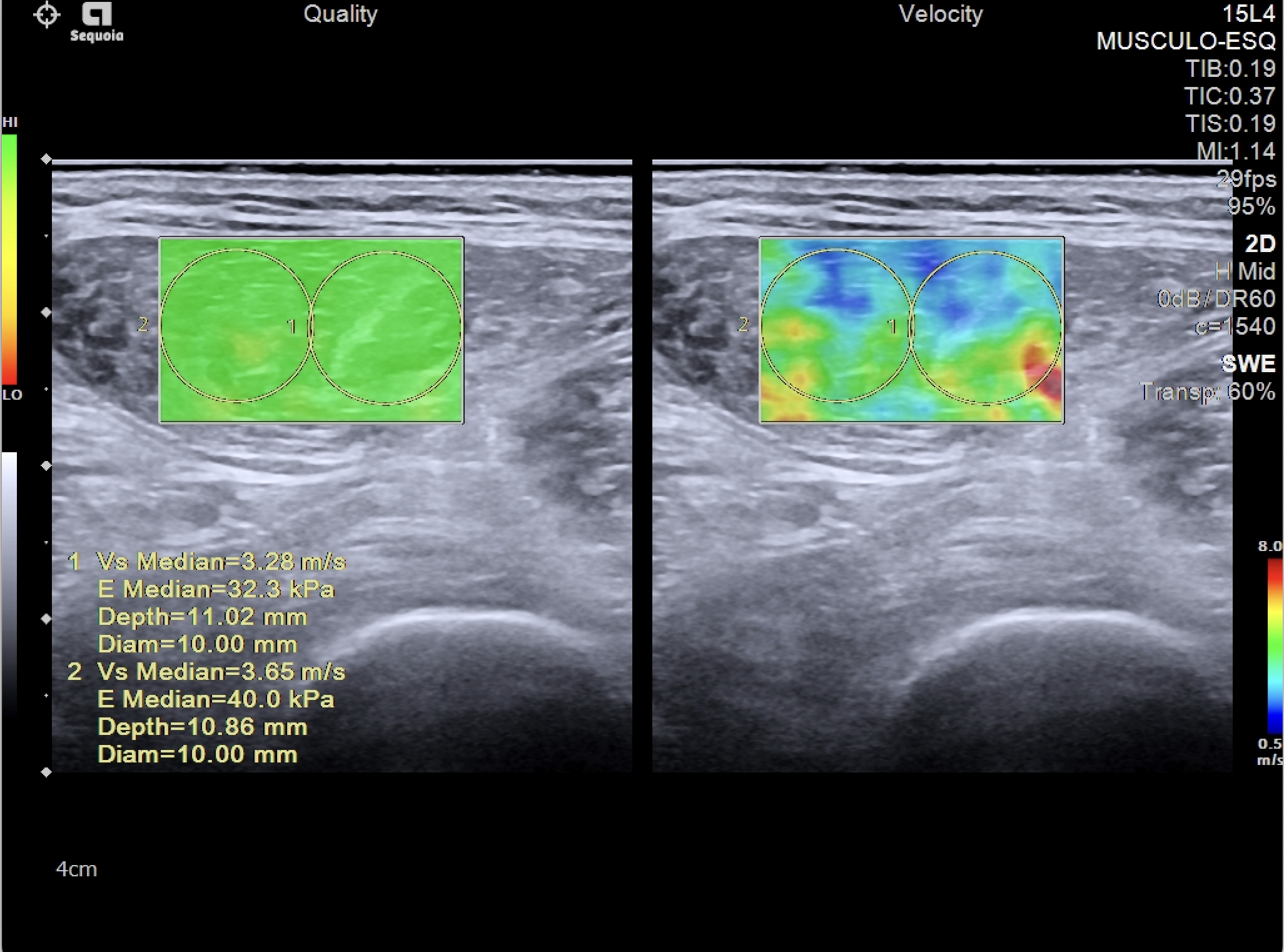
**Figure IV, Sup Material. Ultrasound image of the RF- SWE**.

The figure represents the determination of RF-SWE directly from the ACUSON Sequoia Ultrasound System, Siemens Healthineers ®, with the SWE quality panel (left) and the color map SWE image (right).

**Declarations**

1. **Ethical approval**

**
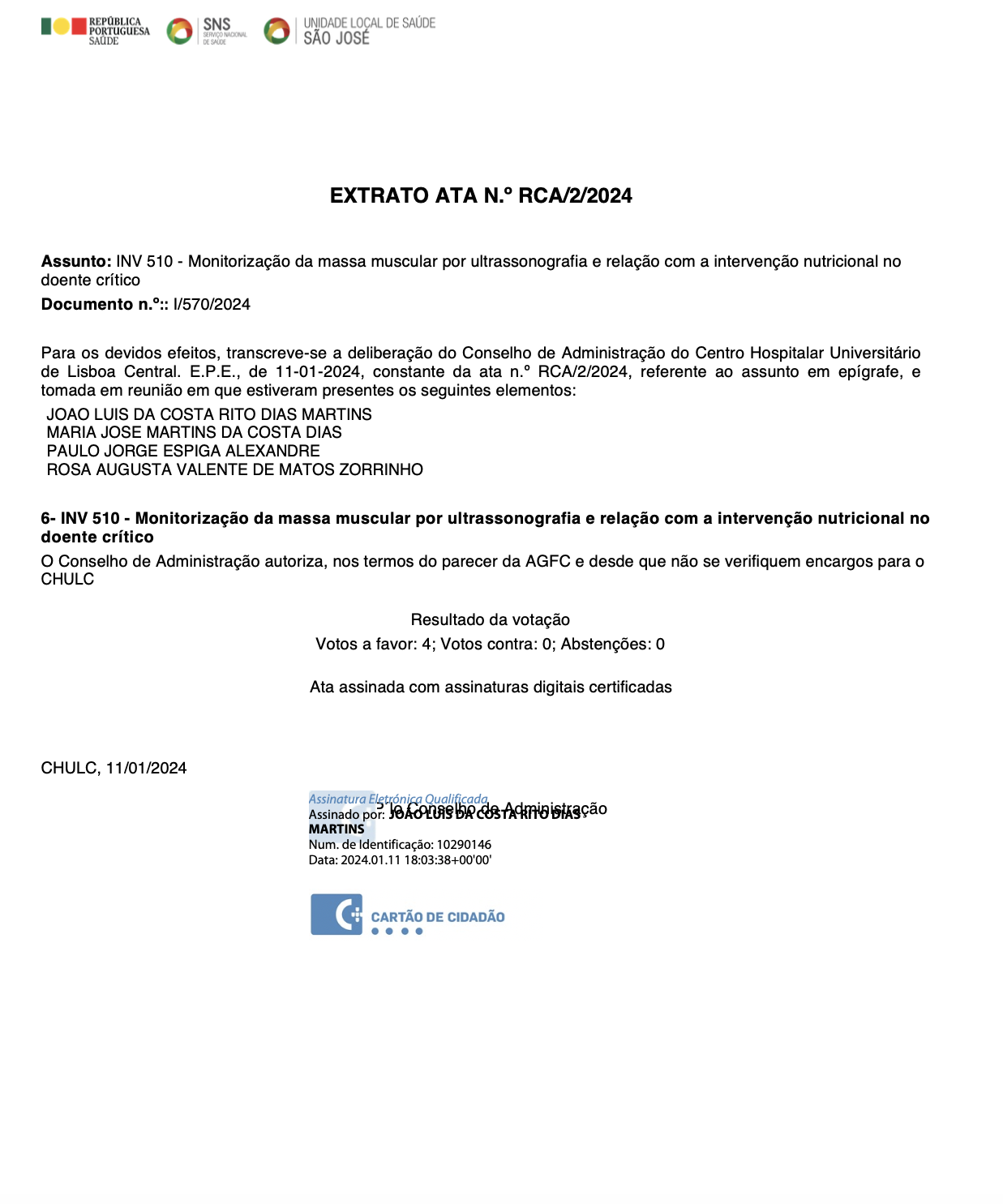
**


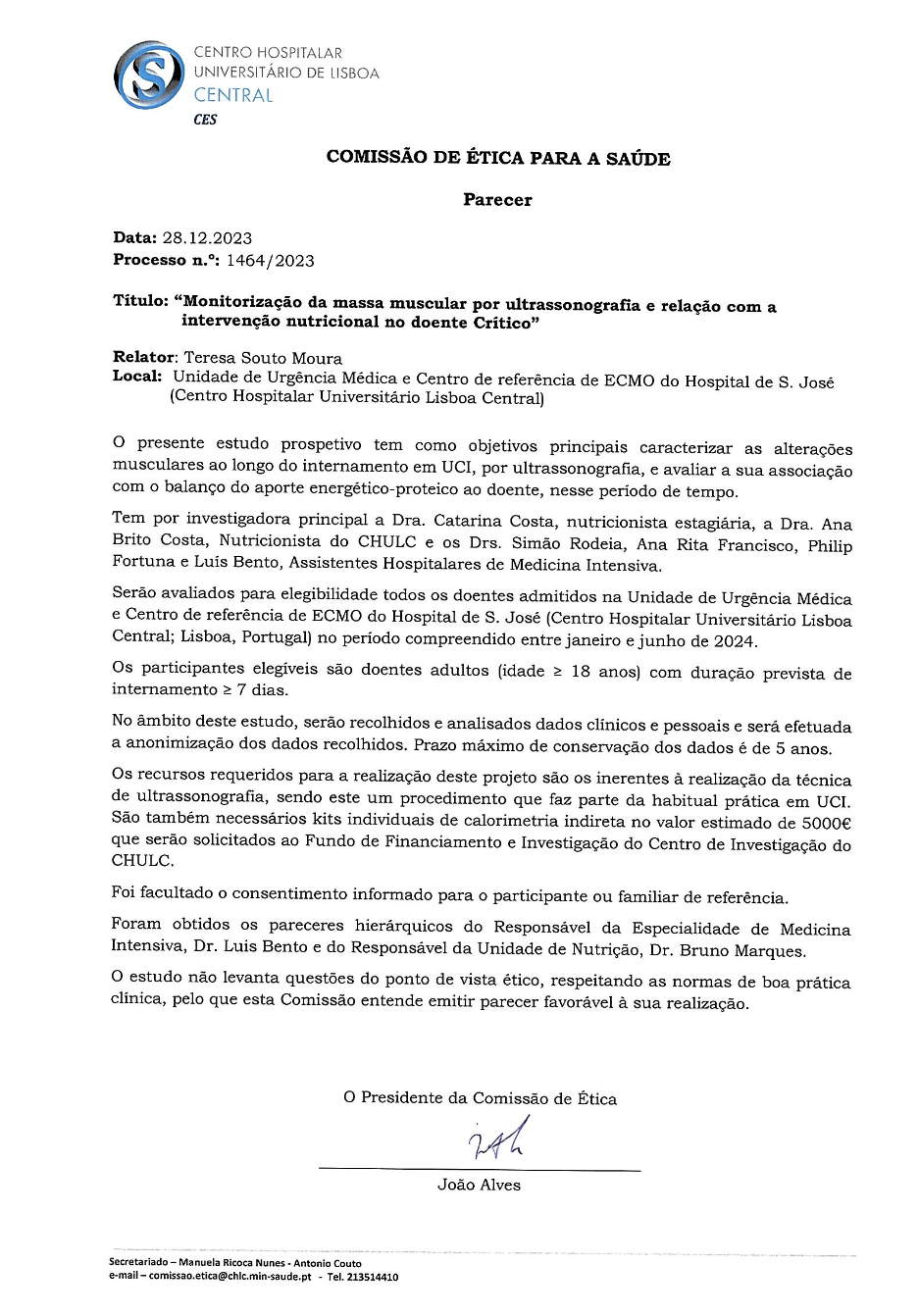


1. **Consent to participate and publication**

**
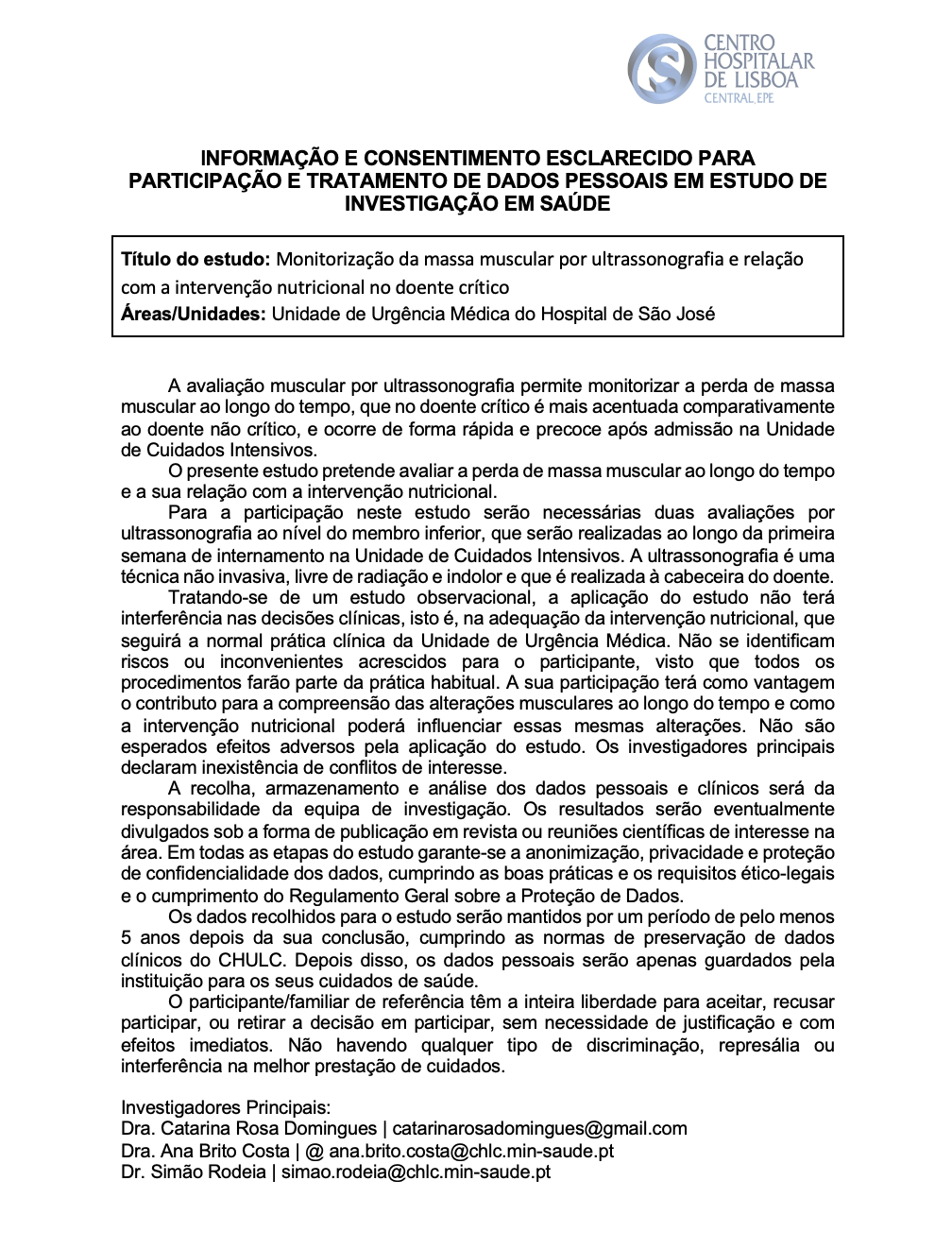
**

**
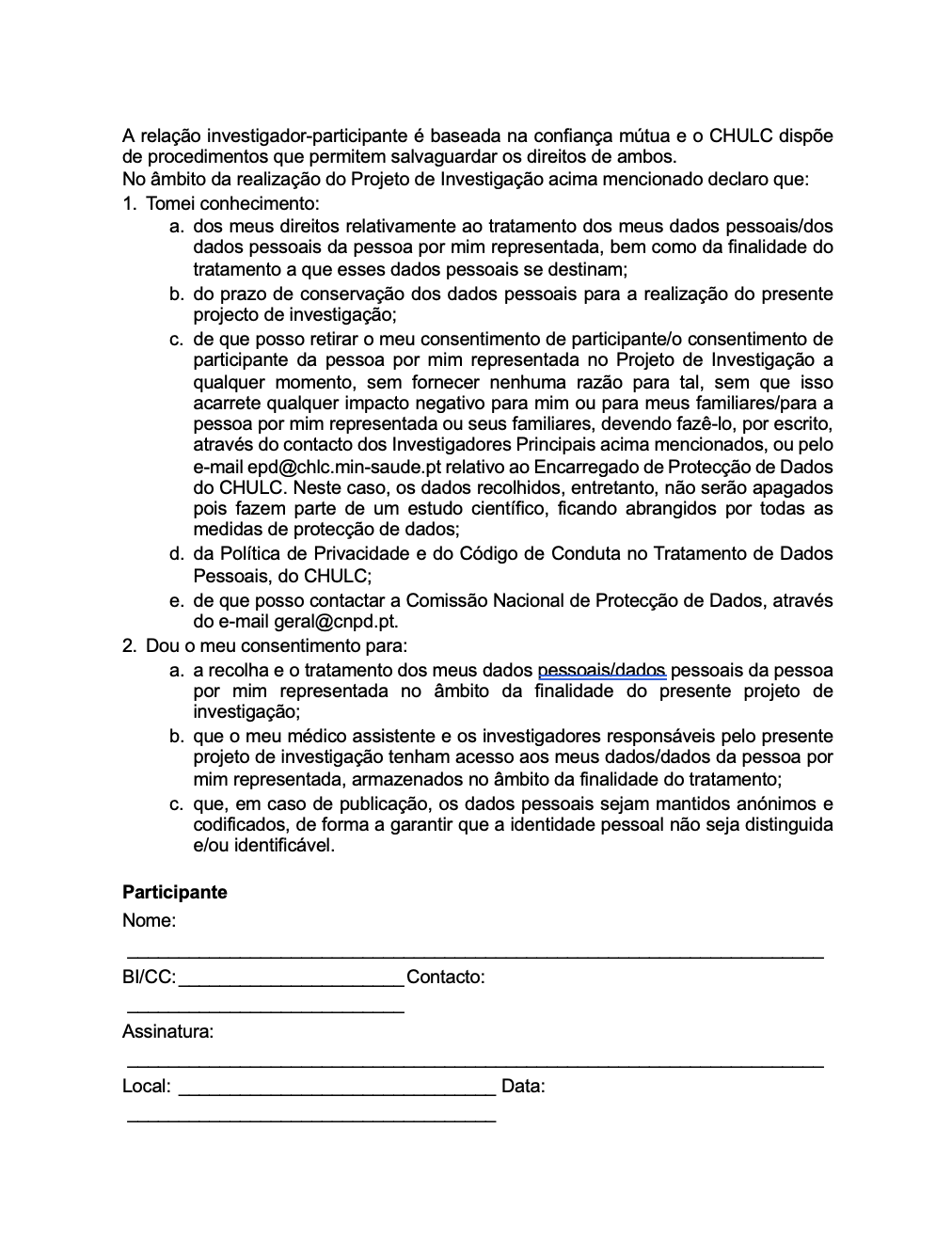
**

**
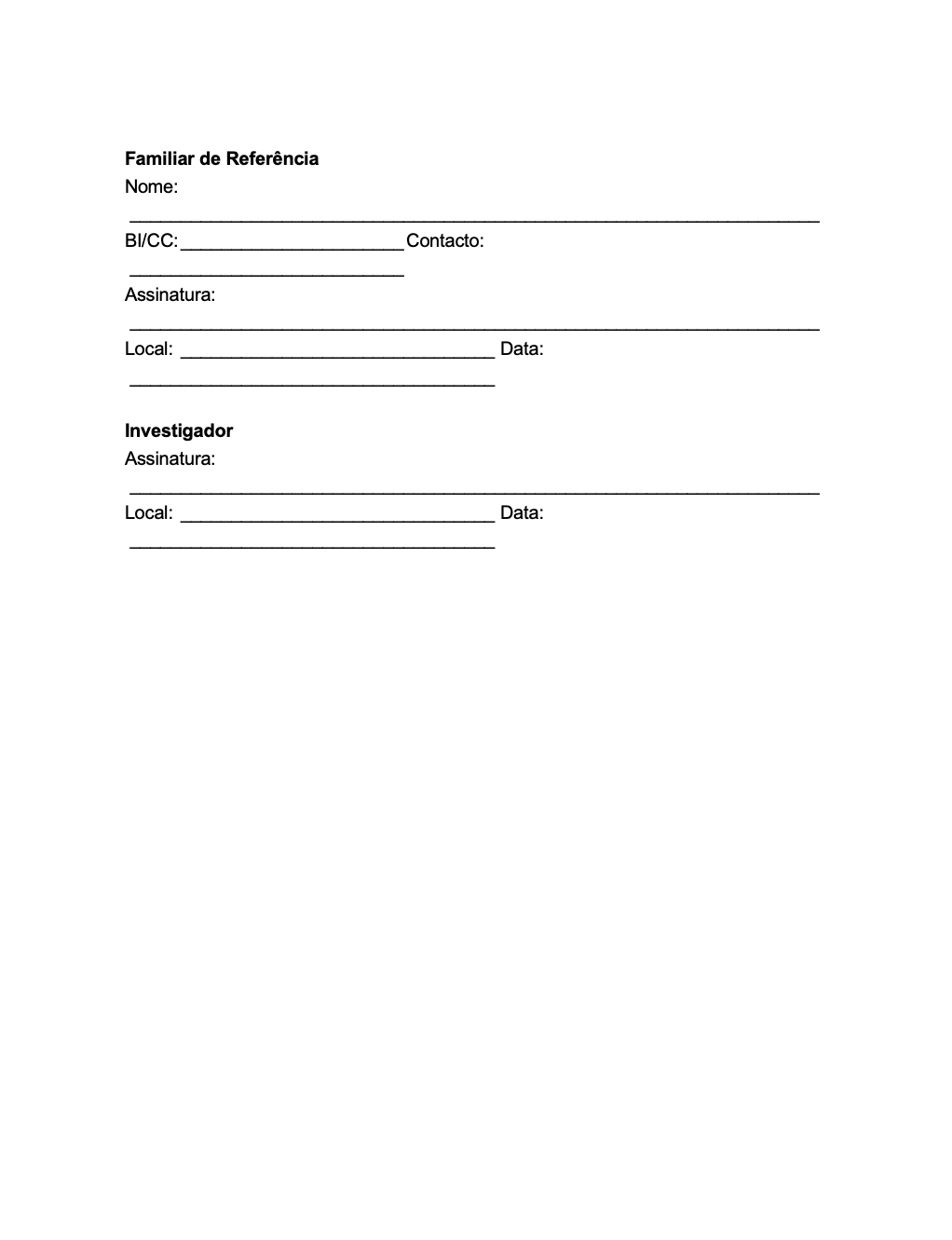
**
